# Nutrient detection by incretin hormone secreting cells

**DOI:** 10.1016/j.physbeh.2011.12.001

**Published:** 2012-06-06

**Authors:** Eleftheria Diakogiannaki, Fiona M. Gribble, Frank Reimann

**Affiliations:** Cambridge Institute for Medical Research and Department of Clinical Biochemistry, Addenbrooke's Hospital, Hills Road, Cambridge, CB2 0XY, UK

**Keywords:** Glucose-dependent insulinotropic polypeptide (GIP), Glucagon-like peptide-1 (GLP-1), Intestinal K-cells, Intestinal L-cells, Nutrient sensing

## Abstract

The hormones glucagon-like peptide-1 (GLP-1) and glucose-dependent insulintropic polypeptide (GIP) are secreted after a meal. Like other enteroendocrine hormones they help to orchestrate the bodies' response to the availability of newly absorbable nutrients and are noteworthy as they stimulate postprandial insulin secretion, underlying what is known as the incretin effect. GLP-1-mimetics are now widely used in the treatment of type 2 diabetes and advantages over older insulinotropic therapies include weight loss. An alternative treatment regime might be the recruitment of endogenous GLP-1, however, very little is known about the physiological control of enteroendocrine responses. This review focuses on the molecular mechanisms to detect nutrient arrival in the gut that have been implicated within the incretin secreting cells.

## Highlights

*►* In vitro and in vivo evidence supports intrinsic nutrient sensing in incretin secreting cells. ► Glucose sensing involves electrogenic sodium coupled uptake via SGLT-1. ► Free fatty acids activate Gq-coupled receptors. ► Elevation of cAMP downstream of Gs-coupled receptors further boosts secretion.

## 1 Introduction

Endocrine cells in the gastrointestinal tract – so called enteroendocrine cells – secrete a range of hormones that regulate glucose homeostasis, gut motility, epithelial proliferation, appetite and adiposity. Two gut peptides, glucose-dependent insulinotropic polypeptide (GIP — formerly known as gastric inhibitory polypeptide) and glucagon-like peptide-1 (GLP-1) are widely recognised for their role as incretins, and underlie the augmentation of insulin secretion that is observed when glucose is administered orally rather than intravenously [1–3]. The incretin effect has been estimated to account for 50–70% of total postprandial insulin secretion [3–5], and although primarily considered as a response to oral glucose, it may also play a physiological role following lipid ingestion [6].

## 2 Properties of the incretin hormones

GIP is a 42-amino acid peptide produced by enteroendocrine K-cells, which are found in highest numbers in the duodenal and jejunal epithelia. It is secreted in response to the intake of fat and glucose [7], with plasma levels rising by 10–20 fold and reaching a peak just 15–30 min after meal ingestion [8]. GIP has a circulating half-life of only a few minutes as it is hydrolysed rapidly by the proteolytic enzyme dipeptidyl-peptidase 4 (DPPIV) into a truncated and inactive product [9]. GIP not only has insulinotropic effects on the pancreatic β-cell but is also implicated in lipid metabolism. Expression of the GIP receptor has been detected on rat [10] and human adipocytes [11], and it has been found that GIP promotes triglyceride incorporation into adipose tissue [12–15], although in human volunteers this was only observed under conditions of hyperglycaemia and did not significantly alter the concentration of circulating triglycerides [12]. Additional evidence demonstrated that GIP can also increase glucose transport and promote fatty acid synthesis in isolated adipocytes [16]. Others, however, have observed lipolytic effects of GIP, as evidenced by enhanced release of glycerol from differentiated 3T3-L1 adipocytes [17].

**Fig. 1:**
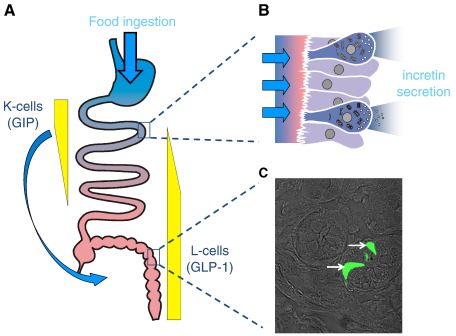
Incretin secreting cells. A) Glucose dependent insulinotropic polypeptide (GIP) is secreted from enteroendocrine K-cells found in highest density in the duodenum, whereas glucagon-like peptide-1 (GLP-1) is secreted from enteroendocrine L-cells found in highest density in the more distal intestine, the ileum and colon. B) K- and L-cells are both “open-type” cells, making direct contact with the intestinal lumen via their apical microvilli, presumably enabling them to directly assess the content composition. Proximally located cells are in an ideal position to assess postprandial nutrient availability, whilst stimulation of more distally located cells probably also involves humoral and/or neural signals (arrow in A). C) Phase contrast image of intestinal cells in a slice through colonic crypts from a mouse transgenic for the yellow fluorescent protein Venus under the control of the proglucagon-promoter. Venus-fluorescence was excited at 480 nm and the two positive cells in the field of view are indicated by the white arrows (photograph by Gareth Rogers).

GLP-1 is a product of alternative processing of proglucagon in enteroendocrine L-cells [18,19], which are found along the length of the intestinal tract but at highest density in the distal ileum and colon [20] (Fig. 1). Following food ingestion plasma GLP-1 concentration rises within minutes and can remain elevated for several hours [21]. Like GIP, secreted GLP-1 is rapidly inactivated by DPPIV [22]. In contrast to the obesogenic properties of GIP, GLP-1 has anorexigenic effects [23]. Exogenous administration of this peptide reduces food intake not only in healthy normal-weight subjects [24] but also in obese humans [25]. It also inhibits gastric acid secretion [26,27], decelerates gastric emptying [28,29] and suppresses glucagon release from pancreatic α-cells [30].

Besides their acute insulinotropic effects, the incretin hormones have additional beneficial effects on the endocrine pancreas. Both GLP-1 and GIP receptors are expressed on pancreatic alpha and beta-cells [31–34] and signalling mediated by these structurally distinct receptors promotes the survival of beta-cell lines [35,36]. Experimental evidence suggests that GLP-1 stimulates beta-cell neogenesis [37] and proliferation, and promotes cell survival in vitro and in vivo [38,39]. A two week long infusion of GIP into diabetic rats resulted in reduced expression of the pro-apoptotic *bax* gene and a parallel increase in the anti-apoptotic *bcl-2* gene [40]. These effects potentially offer an important therapeutic advantage to the incretin hormones compared with other insulin secretagogues used as anti-diabetic agents. Moreover, as plasma GLP-1 levels were found to be decreased in type 2 diabetic patients compared to control non-diabetic subjects [41], the development of novel anti-diabetic interventions that enhance endogenous incretin secretion appears an attractive therapeutic strategy. Incretin based therapies are already available for the management of type 2 diabetes. These include the injectable high affinity GLP-1 receptor agonist exenatide, the DPP4 resistant GLP-1 analogue liraglutide and the orally available DPP4 inhibitors known as the “gliptins”. Specific secretagogues for endogenous GLP-1 secretion from L-cells are not yet available.

## 3 Mechanisms underlying incretin hormone secretion

Enteroendocrine K- and L-cells exhibit a polarised morphology, with an apical surface contacting the gut lumen and a basolateral membrane in close proximity to the circulatory system. This so-called “open-type” morphology should enable them to sense dietary nutrients and non-nutrient substances, present in the intestinal lumen. In live preparations, enteroendocrine cells are not easily distinguished from surrounding enterocytes and thus for many years, attempts to study their stimulus sensing pathways were restricted to the use of perfused intestinal preparations and intestinal model cell lines such as GLUTag, STC-1 and NCI-H716. Until recently, only a few studies worked with primary cells, using either partially enriched cultures isolated from adult tissue of dogs and rats by elutriation [42,43] or foetal intestinal cultures from rats [44]. The recent development of transgenic mice in which GLP-1 or GIP producing cells are labelled with a fluorescent marker has enabled the identification of adult murine L- or K-cell populations in culture, and subsequently the use of single cell recording techniques for the characterisation of nutrient sensing mechanisms in these cell types [45,46]. Both primary L cells and the model GLP-1 secreting cell line GLUTag are electrically active and nutrient responsive [47–49]. Consistent with findings from other endocrine cell types, alterations in membrane potential are coupled via voltage gated Ca^2+^ entry to the release of secretory vesicles. All enteroendocrine cell types studied to date are also responsive to signals that activate G-protein coupled receptors.

## 4 Nutrient sensing

As both K- and L-cells are open-type cells, it seems likely that both can directly sense changes in luminal nutrient concentrations. Some differences between GIP and GLP-1 secretory responses to nutrient ingestion have, however, been observed and it has been proposed that GIP secretion requires nutrient absorption, whilst the mere presence of nutrients in the lumen is sufficient to trigger GLP-1 secretion. This notion is at least in part based on observations that patients suffering from celiac disease or tropical malabsorption, conditions characterised by diarrhoea and inefficient intestinal absorption of nutrients, have markedly diminished postprandial GIP secretion but enhanced or chronically elevated GLP-1 levels (measured at the time as enteroglucagon) [50,51]. Similarly, it has been found that α-glucosidase inhibitors inhibit GIP, but stimulate GLP-1 secretion [52,53]. Another recent report in rats found GIP secretion to be more steeply dependent on luminal carbohydrate dose than GLP-1, irrespective of whether infusion was into the duodenum or ileum [54]. Whilst this may suggest differences in the sensing mechanisms of K- and L-cells, many of the above observations might simply reflect the different distribution of these cells along the length of the gut (see Fig. 1). Any intervention that partially interferes with nutrient absorption, tends also to shift more nutrients into the distal intestine where most of the L-cells are found, and could thereby increase GLP-1 release even if the nutrient sensing mechanism of individual L-cells itself depends on absorption. In the case of glucose, this is illustrated by the differences between GIP and GLP-1 secretion upon duodenal infusion of glucose in human volunteers — whilst GIP secretion was already stimulated by the lowest caloric perfusion rate and increased dose dependently, the mere presence of luminal glucose in the duodenum was insufficient to stimulate GLP-1 secretion, which was only seen at perfusion rates exceeding the duodenal absorption capacity [55]. Differences in GIP and GLP-1 secretory profiles after genetic or pharmacological intervention should thus always take into account the difference in K- and L-cell location. To our knowledge all identified nutrient sensing mechanisms have been found in both cell types, although some differences in the relative contribution of each pathway might exist [45].

## 5 Carbohydrates

Carbohydrates, and glucose in particular, have been the most extensively investigated nutrients in relation to incretin hormone secretion. The majority of studies agree that glucose is a potent secretagogue for both GIP and GLP-1. Other carbohydrates may trigger secretion but are reportedly less effective than glucose. Increased plasma GLP-1 levels have been reported in humans after consumption of glucose but not equivalent portions of complex carbohydrates in the form of brown rice or barley. In a similar pattern, higher levels of GIP were elicited following consumption of glucose than a barley meal [56]. Oral glucose also stimulates GLP-1 secretion more effectively than other monosaccharides such as fructose, although both have been reported to affect appetite similarly [57]. Elevated blood glucose is not a major stimulus for incretin secretion, suggesting that there must be a mechanism for the specific detection of luminal glucose. Several molecular “sensors” have been proposed.

### 5.1 Metabolism and K_ATP_ channel modulation

Initial studies on the mechanisms involved in glucose sensing by enteroendocrine cells were conducted on the model cell line GLUTag. Patch clamp experiments showed that glucose reduced the membrane conductance, depolarised the cell membrane and triggered action potentials. These events were accompanied by elevated levels of intracellular ATP and calcium, and release of GLP-1. Together with the finding that the electrical activity could be abolished by addition of the K_ATP_ channel opener, diazoxide, these results suggested an engagement of K_ATP_ channels in the stimulatory effects of glucose in GLUTag cells [49,58]. Contrary to initial concerns that this may be a cell line artefact, it was subsequently shown that glucose also triggers electrical activity and enhanced GLP-1 secretion in primary cultures of mouse colonic L-cells [46]. K_ATP_ channel subunits, Kir6.2 and SUR1 (sulphonylurea receptor), and the rate limiting glycolytic enzyme, glucokinase, have been detected in GLUTag cells [49] and primary K and L cells from mouse [45,46] or human [59] by quantitative RT-PCR or immunohistochemical analysis. Although initial work on a subclone of STC-1 cells failed to detect GIP secretion in response to glucose [60], studies on mixed epithelial cultures isolated from mouse duodenum demonstrated glucose and K_ATP_ channel dependent GIP release [45].

Although K_ATP_ channels are undoubtedly present and functional in K and L cells, as demonstrated in vitro, closure of these channels is not primarily responsible for linking glucose ingestion to GIP or GLP-1 secretion in vivo. This is supported by the finding that mice lacking the Kir6.2 subunit of K_ATP_ channels exhibited increased rather than reduced levels of GIP secretion in response to an oral glucose load [61]. In humans, the sulphonylurea glibenclamide, which inhibits K_ATP_ channels in β-cells and as a result stimulates insulin release, does not change the levels of GLP-1 or GIP secretion following an oral glucose tolerance test [62]. An analogous conclusion was reached by Pearson et al., who also demonstrated that basal and glucose stimulated levels of GLP-1 and GIP were not different between diabetic patients with mutations in the gene encoding Kir6.2, *KCNJ11,* and healthy control subjects. It is important to note, however, that these patients suffered from neonatal diabetes, characterised by hyperglycaemia resulting from defective sensitivity of β-cell K_ATP_ channels to ATP inhibition which impairs insulin secretion [63].

### 5.2 Metabolism independent mechanisms

Given the evidence above, K_ATP_ channel closure in response to increased glucose metabolism cannot be exclusively responsible for glucose sensing by K and L cells further emphasised by the fact that the non-metabolisable sugars alpha-methyl-glucopyranoside and 3-O-methylglucose have been reported to stimulate GIP and GLP-1 secretion both in vitro and in vivo [45,46,64–66]. Some groups have, thus, postulated that neither metabolism nor uptake is necessary for sugar detection, and that intestinal glucose sensing mirrors lingual sweet taste perception [67]. Two G protein coupled receptors, known as Tas1R2 and Tas1R3, form a heterodimeric sweet taste receptor which recognises glucose and other natural and synthetic sweeteners. Upon ligand activation, a pathway involving alpha-gustducin is stimulated, resulting in stored Ca^2+^ release and subsequent activation of the transient receptor potential channel, TRPM5 [68,69]. Expression of the sweet taste receptor, together with key elements of the signalling pathway such as alpha-gustducin, PLCβ2 and TRPM5, have been reported in human and mouse small intestine and colon [70,71], and colocalisation of alpha-gustducin with the peptides GLP-1 and PYY has been demonstrated immunohistochemically in human L-cells [72–74]. These findings triggered the formulation of a new theory implicating sweet taste receptor signalling in glucose sensing by enteroendocrine K and L-cells, supported by reports that GLP-1 release was stimulated by a range of sweet substances in GLUTag and NCI-H716 cells [74,75] and that alpha-gustducin or Tas1R3 knock-out mice exhibited reduced GLP-1 responses to oral glucose [76]. This theory, however, remains controversial and has been challenged recently by a number of groups. In contrast to experiments in cell lines, studies in healthy humans have shown a lack of effect of sucralose on GLP-1 or GIP secretion [73,77,78], and in type 2 diabetic patients GLP-1 or GIP levels remained unaltered after consumption of the non-caloric sweetener, stevioside [79]. Similar conclusions were reached in in vivo experiments in rodents [80].

**Fig. 2:**
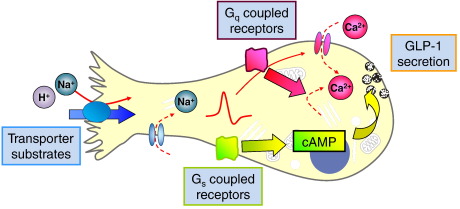
Current model of L-cell nutrient sensing. Nutrients can be taken up by electrogenic transport at the apical pole of the cell (e.g. SGLT-1 for glucose). This directly depolarises the plasma membrane and triggers action potentials, eventually opening voltage-gated Ca^2+^-channels. The subsequent rise in cytosolic Ca^2+^ triggers fusion of GLP-1 containing vesicles. Alternatively intracellular Ca^2+^ might be raised downstream of the activation of G_q_-coupled receptors (e.g. FFAR2/GPR43 for propionate), which would also be expected to activate protein kinase C. Strong stimulation of GLP-1 secretion is, however, also seen upon elevation of cyclic adenosine monophosphate (cAMP), which physiologically would be expected to occur upon stimulation of G_s_-coupled receptors such as GPR119 and GPBAR.

Interestingly, the non-metabolisable sugar analogues that trigger incretin secretion are substrates for sodium/glucose co-transporter-1 (SGLT1), which is responsible for the active uptake of a variety of sugars across the small intestinal brush border membrane. Sugars that are not substrates for SGLT1, such as 2-deoxy-D-glucose and N-acetyl-D-glucosamine, by contrast, did not alter GLP-1 release [81]. Further evidence implicating SGLT1 as a component of the glucose-sensing mechanism derives from the finding that the SGLT1 inhibitor phloridzin reduced glucose triggered incretin secretion both in vitro and in vivo [82–84]. Glucose transport via SGLT1 is a tightly regulated process requiring the contransportartion of two Na^+^ ions per glucose molecule [85]. The role of SGLT1 in glucose sensing mechanism was investigated in the GLUTag cell line where it was demonstrated that the electrogenicity of this transporter induces a glucose-dependent inward current sufficient to depolarise the cell membrane and trigger action potentials [82] (Fig. 2). In GLUTag cells, a high concentration of alpha-methyl-glucopyranoside (100 mM) was necessary to trigger GLP-1 release [82], but in primary cultures much lower concentrations were found to be sufficient, the EC_50_ being 0.2 mM, which is close to the transport Km of heterologously expressed SGLT1 [46]. The importance of SGLT1 driven glucose uptake for incretin secretion was recently emphasised by the absence of GLP-1 and GIP-responses to oral glucose in global SGLT-1 knock-out mice [86], although definitive proof of a direct action of the transporter within enteroendocrine cells has to await a more targeted ablation.

## 6 Lipids

It is well known that fat is a good stimulant for both GIP and GLP-1 secretion [8,87,88]. The response appears to be proportional to the caloric content of the ingested lipid, as secretion is highly sensitive to dose fluctuations [89,90]. In addition to meal size, secretion of the two hormones is affected by the degree of fatty acid saturation, since secretion of GLP-1 in vitro was shown to be preferentially triggered by long chain monounsaturated fatty acids compared with their saturated equivalents [91]. A superior stimulatory effect of unsaturated fatty acids has also been described in humans, as olive oil, which is rich in monounsaturated fatty acids, was found to be more potent in inducing GIP and GLP-1 secretion than butter, which consists mainly of saturated fat [92].

Although fat is reportedly the most effective nutrient stimulus for GIP secretion in humans, carbohydrates may play a more important role in animals such as rodents and pigs [93]. Ingested triglycerides are hydrolysed by pancreatic lipase into free fatty acids and 2-monoglycerides [94]. Liberation of these products is a key event preceding secretion of a number of gut peptides including GIP and GLP-1. This is evident from studies using the lipase inhibitor, Orlistat, which has inhibitory effects on postprandial secretion of GIP [95] and GLP-1 [95,96], although the diminution of the latter is not unanimously accepted [97].

During the last decade, free fatty acids amongst other nutrients, hormones and neurotransmitters have been reported to act as ligands for G-protein coupled receptors (GPCR). Thus, previously orphan GPCRs such as GRP40 (now renamed to free fatty acid receptor 1, FFAR1), GPR41 (FFAR3), GPR43 (FFAR2) and GPR120 have now been demonstrated to be activated by free fatty acids. This discovery led to the assumption that these receptors might be modulators of incretin hormone release, and might, therefore, be promising novel therapeutic targets for diabetes.

GPR120 has been identified in the intestine, principally in K and L-cells [45,46]. Its activation by unsaturated long-chain free fatty acids such as α-linoleate, docosahexaenoeate, palmitoleate and oleate, dose-dependently promoted the secretion of GLP-1 both in vitro and in vivo [98]. Enteroendocrine L and K-cells also express high levels of the related GPCR, FFAR1 [45,46], which serves as a sensor of saturated and unsaturated long-chain fatty acids and has been implicated in the secretion of incretin hormones. Engagement of FFAR1 in the secretory responses is demonstrated by the impaired release of GIP and GLP-1 in FFAR1−/− mice during consumption of a high fat diet [99]. Since both GPR120 and FFAR1 are coupled to G_q_ family proteins, their activation is accompanied by stimulation of PKC and IP_3_-induced calcium release from the endoplasmic reticulum, although other mechanisms have been proposed [100]. According to recent reports, of all the mammalian PKC isozymes, PKC zeta is essential for GLP-1 secretion by long chain unsaturated fatty acids [101]. This particular PKC isozyme is not sensitive to DAG and Ca^2+^ and might thus be recruited independent of G_q_-activation.

Short chain fatty acids such as acetic acid (C2:0), propionic acid (C3:0) and butyric acid (C4:0) are ligands for FFAR2 and FFAR3. The highest expression of these two receptors has been observed in the large intestine [102], which is also the site of synthesis of short chain fatty acids by bacterial fermentation of dietary fibre. FFAR2 couples to Gq and Gi proteins whilst FFAR3 activates mainly the Gi/o family [103]. Immunostaining has revealed specific expression of FFAR2 in GLP-1 containing L-cells of the human and rat colon [104]. Activation of FFAR2 resulted in acute Ca^2+^-responses in primary mouse L-cells, whereas short chain fatty acid dependent GLP-1 secretion was abolished in FFAR2 knock-out mice [105].

To the list of GPCRs, which may function as intestinal lipid sensors coupled to incretin release, should be added GPR119. This is another receptor showing specific expression in K and L-cells along the intestinal tract [45,46,106], and its pharmacological activation by synthetic small-molecule agonists enhanced GLP-1 and GIP levels in mice [106]. One proposed physiological ligand for GPR119 is oleoylethanolamide [107], a lipid amide synthesised in the small intestine during digestion of dietary fat [108]. A more recently identified GPR119 ligand is 2-oleoylglycerol, thus potentially establishing this receptor as a more general sensor of ingested lipid, as 2-monoacylglycerol is a standard intermediate of intestinal triglyceride digestion [109]. In contrast to the FFAR-family, GPR119 is coupled to Gs rather than Gq, and its activation is therefore accompanied by stimulation of adenylyl cyclase and production of intracellular cAMP [110].

Free fatty acids and monoglycerides are absorbed by the enterocytes and re-synthesised into triglycerides in the endoplasmic reticulum [111]. The procedure involves sequential esterification catalysed by the enzymes monoacylglycerol acyltransferase (MGAT) and diacylglycerol acyltransferase (DGAT). The newly synthesised triglycerides are incorporated into chylomicrons, a step controlled by the microsomal triglyceride transfer protein (MTP) before being secreted into the lymph. It has been suggested that chylomicron formation in the small intestine may be associated with the control of gut peptide secretion. This notion is supported by evidence reporting diminished GIP levels following administration of pluronic L81, an inhibitor of chylomicron formation [112]. Furthermore, mice deficient in MGAT2 or DGAT1 displayed reduced GIP release in response to an oral triglyceride load, whilst GLP-1 responses were enhanced in the DGAT1−/− mice [113]. A metabolically unstable MTP inhibitor, developed to inhibit intestinal but not hepatic MTP, has been reported to elevate GLP-1 secretion in rats on a high fat diet, whilst only mildly inhibiting CCK release [114]. The molecular pathways coupling intracellular metabolism of lipids to GIP and GLP-1 secretion have not yet been established and it remains to be seen whether the observed discrepancy between either GLP-1 and GIP responses in the DGAT1 knock-out mice or GLP-1 and CCK responses after pharmacological MTP inhibition simply reflect enhanced delivery of lipids to the more distal gut.

## 7 Proteins

Although the final steps of dietary protein hydrolysis and absorption take place in the small intestine, the role played by protein or individual amino acids in triggering incretin secretion remains an area of controversy. In humans, for example, protein-rich meals were ineffective in altering post-prandial GIP levels [56,115] whereas intraduodenal infusion of mixed amino acids [116,117] or oral consumption of the amino acid glutamine [118] was found to increase GIP release. In animals such as dogs and rats, peptones are considered as potent stimuli for GIP release [119,120]. Similarly, not all groups have confirmed a link between protein rich meals and GLP-1 secretion [54,121]. As for GIP release, a requirement for protein digestion seems likely, as the enteroendocrine cell lines NCI-H716 [122], STC-1 and GLUTag [123] secrete GLP-1 in response to meat hydrolysates, which consist primarily of mixtures of di- and tri-peptides. The amino acid glutamine has also been found to trigger GLP-1 secretion in normal weight and obese human subjects [118].

The primary mechanisms underlying detection of amino acids or small peptides in enteroendocrine cells remain uncertain, as a range of potential signalling pathways have been postulated. Activation of the ERK1/2 MAPK and p38 MAPK pathway has been observed in NCI-H716 cells treated with meat hydrolysate or mixtures of essential amino acids [124], and may provide a link to GLP-1 release. Glutamine promotes the secretion of GLP-1 from rodent primary cultures and GLUTag cells via two pathways. Electrogenic Na^+^ coupled amino acid uptake appears responsible for initiating membrane depolarisation and voltage gated Ca^2+^ entry, whilst a second pathway involves elevation of intracellular cAMP levels [48]. Synergy between these Ca^2+^ and cAMP signalling pathways seems a particularly potent stimulus of GLP-1 release in vitro. The diversity of electrogenic uptake mechanisms for amino acids and dipeptides across the intestinal epithelium, together with the range of G-protein coupled receptors now believed to respond to specific groups of amino acids or small peptides, may provide enteroendocrine cells with a broad repertoire of potential sensors of digested protein *(Tolhurst et al. 2011 Handbook of Experimental Pharmacology 209, Appetite Control, in the press, Ref to be updated once on pubmed)*.

## 8 Conclusion

Luminal nutrients trigger incretin secretion through interaction with a number of different receptors on K- and L-cells. For glucose, it now appears clear that electrogenic uptake through SGLT1 is the primary mechanism coupling GLP-1 secretion to the appearance of glucose in the intestinal lumen. The enteroendocrine cells in this respect appear blind to blood glucose variation simply due to the targeting of SGLT1 to the apical membrane. One prerequisite of this mechanism is that the cells have a relatively low resting membrane conductance, as the currents associated with electrogenic nutrient transport are much smaller than currents achieved by the opening of voltage- or ligand gated ion channels. It would be interesting to establish where exactly on an L-cell sodium- and calcium-carried action potentials [47] are initiated and if there is a targeting of voltage gated sodium channels to an area near the apical membrane making a structure analogous to the axon hillock.

Whilst electrogenic nutrient uptake can initiate incretin secretion by elevating cytosolic Ca^2+^ through voltage gated Ca^2+^-channel activation, more sustained responses are seen when other second messenger pathways are also recruited. Fatty acids seem clearly to activate Gq-coupled receptors, although the secretory responses observed with fatty acids in isolation are fairly moderate and are unlikely to explain entirely the incretin secretory response observed following lipid ingestion. Strong stimulation of GIP and GLP-1 secretion is seen in response to elevation of intracellular cAMP. Gs-coupled receptors like GPR119, which has now been implicated in triglyceride sensing, might thus be important targets in therapeutic stimulation of GLP-1 release. Other Gs-coupled receptors, like GPBAR/TGR5, which is not directly sensitive to nutrients, but is activated by bile acids [125], are likely to play a role in the incretin responses seen after nutrient ingestion and should also provide additional targets for therapeutic stimulation of GLP-1 release.
